# The Long Journey towards Personalized Targeted Therapy in Poorly Differentiated Thyroid Carcinoma (PDTC): A Case Report and Systematic Review

**DOI:** 10.3390/jpm14060654

**Published:** 2024-06-18

**Authors:** Odysseas Violetis, Panagiota Konstantakou, Ariadni Spyroglou, Antonios Xydakis, Panagiotis B. Kekis, Sofia Tseleni, Denise Kolomodi, Manousos Konstadoulakis, George Mastorakos, Maria Theochari, Javier Aller, Krystallenia I. Alexandraki

**Affiliations:** 1 2nd Department of Surgery, Aretaieio Athens Hospital, Medical School, National and Kapodistrian University of Athens, 11527 Athens, Greeceaspyroglou@gmail.com (A.S.);; 2 Endocrine Surgery Department, Athens Medical Center, 15125 Athens, Greece; p.kekis@hotmail.com; 3 Department of Pathology, Medical School, University of Athens, 11527 Athens, Greece; stseleni@med.uoa.gr; 4 European Neuroendocrine Tumor Society (ENETS) Center of Excellence, Ekpa-Laiko Center, 11527 Athens, Greece; denisekol.dk@gmail.com; 5 IATROPOLIS Private Medical Center, 11521 Athens, Greece; 6 Department of Oncology, Ippokrateio Athens General Hospital, Medical School, National and Kapodistrian University of Athens, 11527 Athens, Greece; mtheochari@gmail.com; 7 Endocrinology Department, Hospital Universitario Puerta de Hierro Majadahonda, 28222 Madrid, Spain; javier.aller@salud.madrid.org

**Keywords:** poorly differentiated thyroid carcinoma, hobnail thyroid carcinoma, tall-cell thyroid carcinoma, molecular-targeted therapy in thyroid cancer

## Abstract

Background: Poorly differentiated thyroid carcinoma (PDTC) has an intermediate prognosis between indolent well-differentiated thyroid carcinoma (TC) and anaplastic carcinoma. Herein, we present a case report with a PDTC component, along with a systematic review of the literature. Case Report: We report a case of a 45-year-old man diagnosed with a PDTC component, along with hobnail and tall-cell variant features positive for BRAFV600E mutation, after a total thyroidectomy and neck dissection. Radioactive iodine (RAI)-131 therapy was applied, but an early recurrence led to complementary surgeries. The anti-Tg rise, the presence of new lymph nodes, and the negative whole-bodyradioiodine scan were suggestive of a radioiodine-resistant tumor. Lenvatinib, sorafenib, dabrafenib/trametinib, cabozantinib and radiotherapy were all administered, controlling the tumor for a period of time before the patient ultimately died post-COVID infection. Systematic Review: We searched PubMed, Scopus, and WebofScience to identify studies reporting clinicopathological characteristics, molecular marker expression, and management of non-anaplastic TC with any proportion of PDTC in adult patients. Of the 2007 records retrieved, 82were included in our review (PROSPERO-ID545847). Conclusions: Our case, together with the systematic review, imply that a combination of molecular-targetedtreatments may be safe and effective in patients with RAI-resistantBRAF-mutated advanced PDTC when surgery has failed to control tumor progression.

## 1. Introduction

Nowadays, thyroid cancer (TC) is estimated to cause approximately 44,000new invasive cancer cases, with a higher prevalence in females [1]. Until recently, it was deemed one of the fastest cancer entities [2,3,4,5], largely as a consequence of oversurveillance and overdiagnosis of small, differentiated thyroid carcinoma (DTC) [3], attributed to significant developments in diagnostic imaging techniques [4,6,7]. However, the adoption of more restrictive criteria for the diagnosis of TC led to a drop in incidence by 2% since 2014. Five-year survival has remained generally stable over the years, being more than 92% in the United States [1]. DTC, including papillary thyroid cancer (PTC) and follicular thyroid cancer (FTC), accounts for more than 90% of TC [8]. De-differentiated TC is rare, classified as poorly differentiated thyroid cancer (PDTC) and anaplastic thyroid cancer (ATC), with reported incidence of 2–15% and 1.7%, respectively [9,10]. A PDTC prognosis is intermediate between DTC and ATC [9], while ATC is associated with the highest mortality risk of any thyroid-arising tumor [11,12].

Most DTC patients have a good prognosis, with a 5-year survival reaching 99% [1,13,14,15,16]. However, at the other end of the spectrum, some PTC variants are associated with aggressive behaviors, such as the recently described hobnail variant, the tall-cell variant, the columnar variant, the solid-trabecular variant [17,18,19,20,21,22,23], the diffuse sclerosing variant [24], the cribriform-morular variant (associated with FAP and APC gene alterations) [25,26], and the oncocytic variant [27]. Approximately 10–15% of all TCs exhibit aggressive behavior and high disease-specific mortality [28]. Among the array of genetic mutations identified in different histologic subtypes of TC, BRAFV600E has been extensively studied because it appears as the most common driver mutation in thyroid PTC, while PTC without BRAF mutations mostly harbors RAS mutations. The presence of BRAF or RAS mutations dictates significant assets of TC. Sequential acquisition of key genetic alterations promotes progressive dedifferentiation to more advanced TC, namely high-grade differentiated thyroid carcinoma (HGGTC), PDTC, and ATC [29]. Monotherapy with a BRAF inhibitor (BRAF-I) may result in temporary clinical remission, but acquired resistance is frequently observed, leading to significant relapse in the majority of cases [30]. The combination of BRAF-I and MEK inhibitors (MEK-I) in conducted clinical trials has shown delay in the development of resistance mechanisms and was recently approved by the Food and Drug Administration (FDA) as a therapy for BRAFV600E-mutant ATC [31].

We describe herein a patient with multiconstituent TC with poorly differentiated, hobnail, and tall-cell components, ab initio. The patient demonstrated refractoriness to radioactive iodine (RAI) treatment, was treated with tyrosine kinase inhibitors (TKI) and BRAF-I/MEK-I combination, and rechallenged with TKI with established disease control. Because of sparse clinical data and a lack of explicit management guidelines, we also conducted a systematic review of the literature regarding PDTC cases to compare the different approaches followed for this rare yet aggressive entity and to correlate them with our case. Herein, histopathological and clinical features, along with treatment and genetic profiling of TC with any component of PDTC, are addressed.

## 2. Materials and Methods

Aiming at presenting demographics and histopathological and molecular features of PDTC, we conducted a systematic review of the literature scrutinizing three databases, namely PubMed, Scopus, and Web of Science. This systematic review was performed following recommendations of the Preferred Reporting Items for Systematic Reviews and Meta-Analyses (PRISMA) [32]. Without limitations based on the type of study (case reports, case series cohort studies, case–control), adult patients with non-ATC with any proportion of PDTC from the year 2017 to April 2024 were included in the present study, whereas studies in pediatric populations or those lacking relevant demographic, histopathological, or management data or data derived from animal models were excluded. Cases presenting with any component of ATC were also excluded. Review articles, conference abstracts, and opinion articles were not included in the present study either. Since there are differences in the diagnostic criteria of PDTC communicated in the bibliography, we did not use specific criteria for the diagnosis and relied on the PDTC characterization of each study. Although PDTC has been recognized as a distinct pathologic entity based on the WHO classification since 2004, the 4th WHO classification only adopted specific unanimous diagnostic criteria (Turin criteria) in 2017, and they indicated that any poorly differentiated component should be mentioned in the pathology report. Thus, we decided to narrow our search to the period of 2017–2024. The clinicopathological characteristics included in our review consisted of age at diagnosis, gender, histological subtypes, nodal status, distant metastases, extrathyroidal extension, molecular subtypes, surgical performance, the administration of radiotherapy and/or chemotherapy, and outcomes. We used the following terms for our search in the abstract or title areas: poorly differentiated, thyroid cancer/neoplasm/carcinoma, PDTC. Boolean operators (AND, OR) were also used to narrow down the search.

Two independent reviewers (O.V., A.S.) screened the titles and abstracts of the articles and selected potentially relevant studies. Full-text articles were then studied, and the final articles were selected based on the inclusion and exclusion criteria. Disagreements in the review process were resolved through discussion and judgment by a third reviewer (K.I.A.). All studies were carefully compared to avoid the inclusion of duplicate or overlapping samples. Any duplicates identified were removed using EndNote 21. The systematic review is registered in the international prospective register of international reviews, PROSPERO (ID 545847). This study was approved by the local institutional ethics committee with reference number 363/13-10-2021.

## 3. Case Presentation

A 45-year-old man was referred to our department after undergoing a total thyroidectomy with central and lateral lymph node dissection in October 2012 followed by 80mCi of RAI treatment four weeks postoperatively in November 2012 with post-iodine uptake only in the thyroid bed. The pathology report described a PTC of the left lobe with a tall-cell variant, a maximum diameter 5 cm with extrathyroidal extension (ETE), and metastases in three out of 28 resected lymph nodes (isthmus area and transition area between isthmus and left lobe). The TNM status was Τ4Ν1aΜ0 (Stage ΙΙI) [33], which along with the tall-cell variant, placed the patient at intermediate risk. Thus, he was referred to our clinic, and the levothyroxine dose was titrated to achieve a suppressed thyroid-stimulating hormone (TSH). His past medical history was remarkable for hypercalciuria on hydrochlorothiazide, nephrolithiasis, chronic sinusitis, and positive thyroid-stimulating immunoglobulin (TSI) without documented thyroid dysfunction prior to the thyroidectomy. The patient reported no family history of thyroid disease, malignancy, or personal history of radiation exposure.

**Figure 1:**
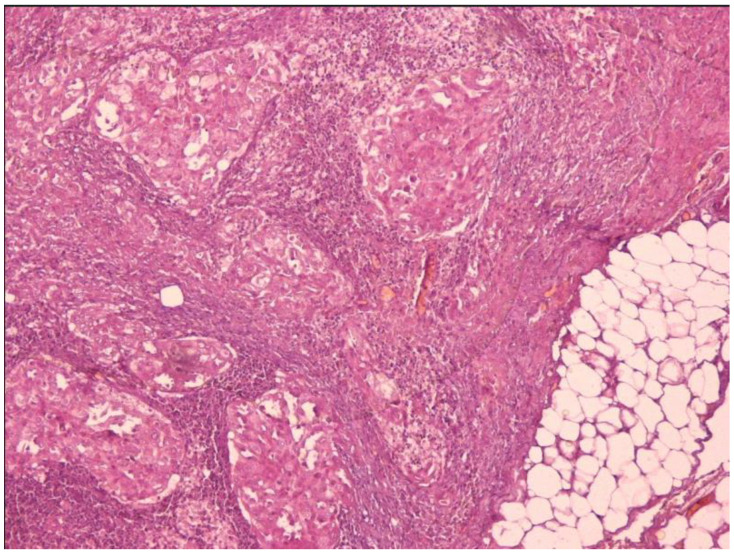
Invasive front with a solid pattern and nuclear atypia (HE × 250).

A documented increase in anti-Tg levels and a suspicious left neck lymph node enlargement, revealed by an ultrasound scan, were suggestive of disease recurrence. In March 2013, the patient underwent a complementary left lateral lymph node dissection, with 1/11 lymph nodes positive for the aforementioned carcinoma. In June 2014, diagnostic WBS after recombinant thyrotropin alfa stimulation was performed, and one lymph node 3.7 cm in the left compartment was visualized, along with a further rise in anti-Tg title (1081 IU/mL; Tg < 0.04 ng/mL; TSH 130.7 μIU/mL) under maximal stimulation. The stimulation with thyrotropin alfa was repeated during 18F-FDG-PET/CT, and no further uptake was evident other than the described lymph node. An ultrasound (US) scan and neck magnetic resonance imaging (MRI) scan pinpointed two additional heterogeneous 5–10 mm lymph nodes, as well as a 8 × 5 mm left submandibular lymph node with central vascularization. At this stage, the patient agreed to review the first histology, where a component of PDTC was seen (Figure 1), along with hobnail and tall-cell variant features, as previously reported [34]. Importantly, the molecular and immunohistochemical analysis of previous biopsies showed a positive BRAFV600E mutation (exon 15, c.1799 T > A [p.V600E,COSM 476]). The metastatic focus pathologic review showed a histological pattern reminiscent of the differentiated component. Subsequently, the patient was submitted to a third surgical intervention and underwent a left lateral lymph node dissection in July 2014 with resection of one large metastatic lymph node and 14 lymph nodes free of disease, while the pathology report reconfirmed the origin from the differentiated neoplastic component. An additional RAI treatment dose of 150 mCi was administered in October 2014. A decline in anti-Tg levels was temporarily observed, but later, anti-Tg levels progressively increased, while a suspicious lymph node became apparent in the US in September 2015. The WBS did not show a clear uptake, suggesting RAI refractory (RAIR) disease (anti-Tg 222.6 IU/mL; Tg 0.079 ng/mL; TSH 120.9 μIU/mL), and the patient was submitted to a fourth surgery in November 2015 with deep central compartment dissection of the suspicious lymph node (1/1). Histology once again was consistent with the original differentiated component, with hobnail features staining positive for thyroglobulin and galectin. The follow-up US in May 2016 revealed another infiltrated lymph node internally from the upper part of the left carotid artery and jugular vein 17 × 11 × 19 mm, which increased to 23 × 30 × 30 mm in the subsequent US in September 2016. A fifth surgery took place in October 2016, with complementary left lateral dissection and removal of the large lymph node and two smaller lymph nodes in the bulb of the common carotid artery, all infiltrated by the neoplasm with the tall-cell variant features. However, 3 months later, another suspicious lymph node was seen along the left jugular vein, measuring 7 × 4mm. The next US in June 2017 showed an increased number of infiltrated lymph nodes in the left side levels IIα, ΙΙβ, ΙIΙ, and IV, and a fine needle aspiration confirmed lymph node metastases from the hobnail variant. 

**Figure 2:**
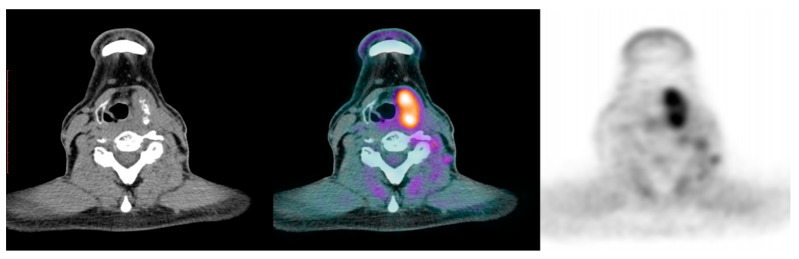
Increased 18-F FDG uptake in the left sphenoid sinus. First image: CT scan, second image: merged CT and PET scan, third image: FDG uptake.

At that point, in July 2017, therapy with full-dose lenvatinib was initiated. As early as within the first month of lenvatinib treatment, our patient presented adverse effects (AEs, according the CTCAE Version 5.0) in the form of palmar–plantar erythrodysesthesia syndrome and abdominal distension. At the completion of 12 months of treatment, he also reported diarrhea (grade 1), fatigue, severe headaches lasting from one to two days per week (all grade 2), and arterial hypertension (grade 2) that prompted the initiation of antihypertensive treatment in April 2018. Because of the headaches fluctuating in severity between grade 2 and 3 and the stable disease course, he was switched to full-dose sorafenib. However, as he soon manifested additional AEs, one week later, he restarted treatment with lenvatinib for a total period of 22 months. In the meantime, the infiltrated lymph nodes increased, and he received radiotherapy in the form of tomotherapy (Helical Tomo therapy along with daily image-guided radiation therapy) from January to March 2019 (33 sessions cyber knife: total dose 66 Gy; daily dose 2 Gy), resulting in a progressive size reduction of the infiltrated lymph nodes. In January 2020, because of a persistent headache, a brain CT was performed. Two hypervascular lesions were seen in the parietal and occipital lobe without edema, suspicious for metastatic foci, along with an increase in the size of the sphenoid sinus due to a heterogeneous material (suspicious for malignant invasion in the sphenoid sinus). These suspicious findings prompted earlier restaging. The ^18^F-FDG-PET/CT was extensively positive, revealing increased uptake in the thyroid bed (SUV:11.4), uptake in the lymph nodes in the levels of IIβ (SUV max 7.7), Vβ (SUV max 3.2) of the left neck, in the liver (SUV max 8), the left sphenoid sinus with extension to the right (SUV max 18.7), cervical vertebra C5 (SUV max 7.8), and laterally to the thyroid cartilage (SUV max 13.4) (Figure 2). All the suspicious areas were confirmed using a brain MRI, an abdominal CT, and bone scanning. Thus, the patient received cyber knife therapy for the brain metastases and conventional radiotherapy for the cervical vertebra metastatic focus. For the later finding, he received 120 mg of denosumab monthly for 3 months.

**Figure 3:**
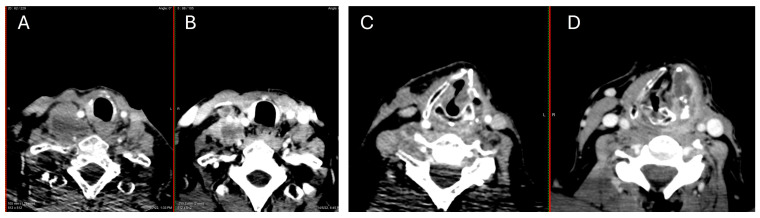
Response to cabozantinib treatment: differentiation of right supraclavicular lymph block before (**B**) and after (**A**) treatment; metastatic foci size reduction before (**D**) and after (**C**) treatment, respectively.

**Figure 4:**
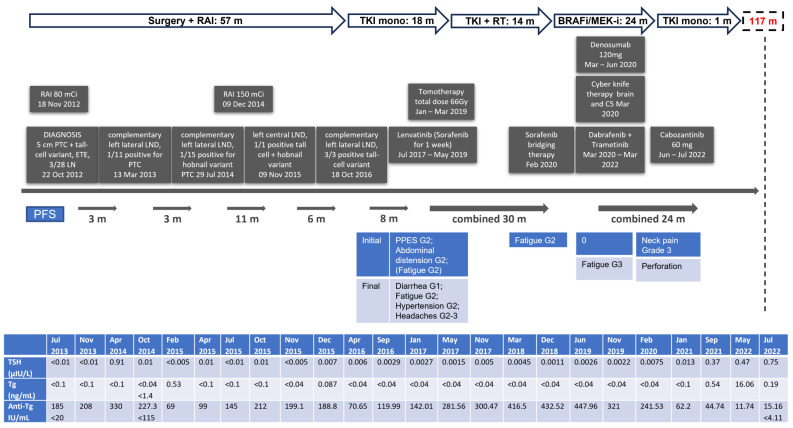
Patient timeline. Arrows depict the time frame of each treatment and the cumulative treatment. The respective PFS is depicted in months (m). anti-Tg: anti-thyroglobulin antibodies, G: grade, LND: lymph node dissection, mono: monotherapy, PFS: progression-free survival, PPES: palmar–plantar erythrodysesthesia syndrome, PTC: papillary thyroid carcinoma, RAI: radioactive iodine, RT: radiotherapy, Tg: thyroglobulin, TKI: tyrosine kinase inhibitor.

According to recent clinical data [35,36], and taking into account the intolerance and the progressive resistance to multi-kinase inhibitor treatment (lenvatinib, sorafenib) in our case, the patient received a therapeutic trial with a combination of BRAF-I/MEK-I in April 2020 with sorafenib as a bridging therapy until the approval reporting fatigue (grade 3). Conventional doses were well tolerated by our patient (dabrafenib 150 mg PO BID, trametinib 2 mg PO QD). Repeated brain and neck MRIs in July 2020 showed radiological improvement with shrinkage of the brain lesions, the left sphenoid sinus, and the area surrounding the thyroid cartilage. Upon subsequent imaging in January 2021, the abdominal MRI was unremarkable, while significant improvement was noted in the brain MRI, with a decreased size of both the occipital (from 6 mm to 5 mm) and parietal (from 20 mm to 8 mm) lesions, a decreased size of the sphenoid sinus, and no new foci. The follow-up neck CT in September 2021 showed a decreased size of the lymph node in the left compartment at the posterior border of the sternocleidomastoid muscle (from 1.3 × 1.7 cm to 1.6 × 2 cm) and a stable size of a lymph node 1 cm in the right submandibular area. Quite a few lymph nodes in the submental area, the right submandibular area, and along the right internal jugular vein remained stable in size as well. Initially, the dabrafenib/trametinib combination treatment was well tolerated, but the patient discontinued his treatment due to fatigue later on. A new brain CT in April 2022 showed edema in the lower part of the parieto-occipital area and in the right frontal area. The neck MRI showed a mass invading the left larynx and the median line with extension to the hypopharynx. In parallel, an increase in the number and size of the lymph nodes of the left neck was seen, together with the presence of a new lymph node in the supraclavicular area. An attempt to obtain a biopsy from this mass failed. A frozen biopsy from the latest operation in 2016 was sent for molecular analysis, but no alteration was detected in genes NTRK1, NTRK2, NTRK3, or RET. In May 2022, and without being on any therapeutic scheme, the patient was admitted to the hospital with acute dyspnea, and a laryngeal edema was documented, which was controlled by a tracheotomy. Subsequently, treatment with 60 mg of cabozantinib was decided on the basis of the COSMIC-311 trial [37], and an objective improvement was already evident one-month post-therapy (Figure 3). The patient complained of persistent pain and discomfort at the site of the tracheotomy, which was removed according to his request. However, the patient was then diagnosed with COVID-19, and he was admitted to the hospital in the COVID unit. During his admission, he refused to have an angiography to further clarify a small hemorrhagic area in his neck identified by the neck surgeons, probably due to perforation (grade 4) caused by the treatment. He signed to be discharged against medical recommendations and died one week later at home due to hemorrhage. The treatment timeline of the patient is also depicted in Figure 4.

## 4. Systematic Review Results

**Figure 5:**
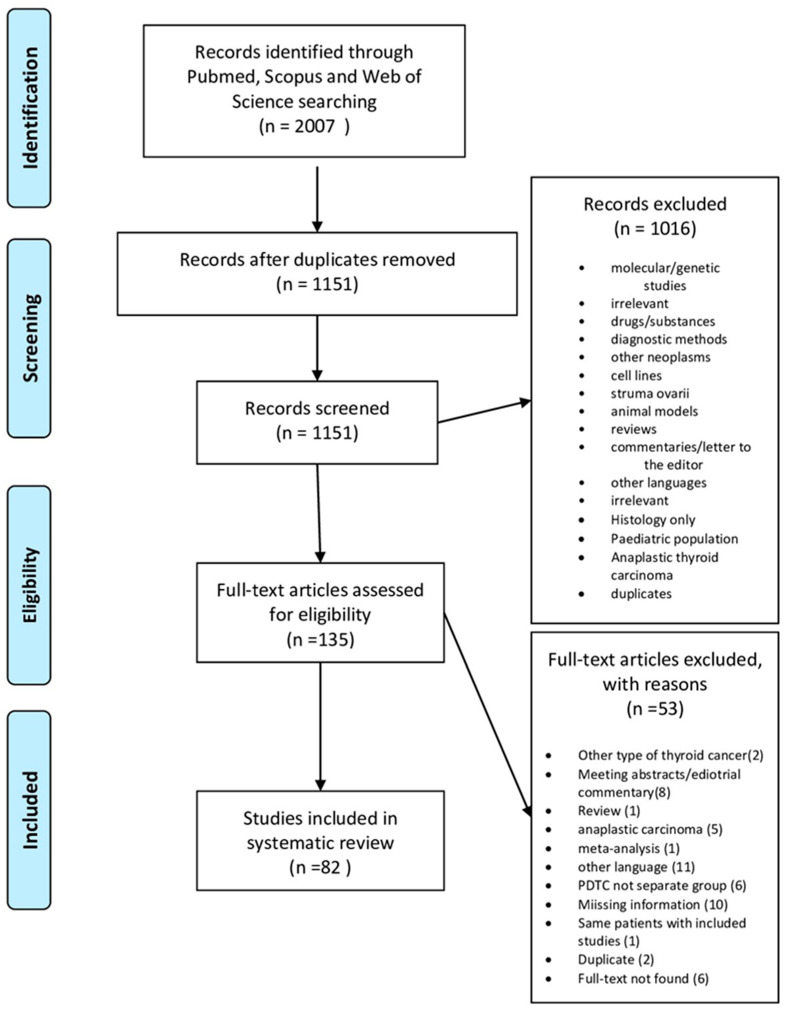
PRISMA flow diagram.

The electronic search from the databases yielded 2007 candidate studies, out of which 1872 were excluded (duplicates, reviews, title or abstract irrelevant to the inclusion criteria), leaving 135 studies for full-text review. After the full manuscript consideration, we finally included 82 studies fulfilling the inclusion criteria (Figure 5). The characteristics of the included studies are shown in Table 1. The excluded studies, along with the reasons for their exclusion, are shown in Figure 5.

## 5. Discussion

The present case shows that the natural history of a non-classic multi-component PTC may be dictated by the more aggressive histological phenotype. The early conversion into a RAIR tumor may also be predictive of shorter survival. The stepwise treatment approach has to be individualized based on molecular and clinical features owing to the lack of large series to guide the best treatment sequence. In the present systematic review, several characteristics of PDTC, such as clinicopathological traits and treatment options, are covered and discussed based on a total of 82case reports and cohort studies found in addition to our case.

### 5.1. Age and Sex

PDTC represents a rare but aggressive cancer subtype originating from thyroid follicular cells and making up approximately 2–7% of all TC [120]. Our systematic review has confirmed the limited data on PDTC cases, as only 82articles were included in our review, and so far, few cohort studies have been conducted. The age range registered herein is 12–93 years, with the majority of the patients being older than 50 years. We observed similar incidences between male and female patients, with a slight female predominance in a few cohort studies. Generally, PDTC is more common in adulthood, with a mean patient age of 60 years; though, cases of young patients have also been described [121]. The disease shows a slight predominance in females, which is concordance with DTC incidence in women [122].

### 5.2. Surgery and RAI Treatment

Since cytological diagnosis of PDTC based on FNAC is challenging, our review has shown that some patients underwent a hemithyroidectomy or subtotal thyroidectomy with a completion thyroidectomy after histological diagnosis [38,44,52,54,59,65,69,85,103,104,107,108,115,117], but the majority of them were submitted to a total thyroidectomy as the initial operation [40,41,42,43,46,48,49,50,51,53,54,55,56,58,60,61,62,64,67,68,69,72,73,75,79,80,81,82,85,86,88,89,90,91,92,93,95,96,100,101,102,103,106,107,108,109,110,112,113,117]. Lymph node dissection was applied in most patients [40,41,42,46,47,49,50,58,61,62,67,71,73,74,75,77,78,79,80,81,82,94,95,96,99,100,101,102,103,106,107,109,110,112,114,117], but the general philosophy was to carry out central or lateral neck dissection, provided that clinical or radiological enlargement of the nodes was evident. Accordingly, in several of the revised cases, sequential local resections were applied, depending on the respective recurrence.

While surgical resection remains the mainstay of treatment, the role of RAI remains controversial. PDTC as an intermediate between DTC and ATC may retain its ability to express thyroglobulin and uptake radioiodine, reflecting its partially well-differentiated features. Compared to DTC, PDTC does not usually respond to therapy, despite partial iodine avidity. Given the higher rate of ETE, R1 surgery, lymph node positivity, and distant metastases, adjuvant treatment should be considered. Sanders et al. [9] recommended considering adjuvant RAI in all PDTC patients, thereby gaining a potential benefit without risking significant morbidity. However, despite the RAI avidity in a high percentage of PDTC cases, no significant impact on survival has been reported after RAI treatment. Unlike in DTC [123,124], RAI appears to be relatively ineffective in the control of distant metastases in PDTC; thus, it is reasonable to consider the use of RAI for metastatic/advanced disease that demonstrates definite iodine uptake [125,126]. Likewise, most cases in our review received at least one cycle of RAI treatment, even without detectable metastatic foci [38,39,40,41,42,43,46,48,49,50,51,53,54,55,58,59,60,61,62,63,64,65,66,67,68,69,73,74,75,80,81,82,85,86,87,88,90,91,97,99,101,102,104,105,106,107,108,109,110,111,112,113,115,117]. In our case report, RAIR disease was evident after two RAI treatments. External beam radiation therapy (EBRT) is a viable option for controlling local disease in patients with PDTC and was utilized in the subset of patients with unresected, locally advanced disease or with metastatic foci such as bone lesions [42,49,50,58,60,61,62,63,64,67,68,69,70,74,75,80,81,85,90,91,97,98,102,106,107,108,109,112,113,116].

### 5.3. Histology

Our patient’s tumor histology was confirmatory of concurrent PDTC, hobnail, and tall-cell variant, all supportive of its aggressive nature, while all lymph node metastases from the first half of the long journey of this disease originated from the differentiated component of the tumor. The aggressive concomitant histological subtypes present in our case, hobnail PTC is a particularly rare variant (approximately 1% of PTC) [19], with frequent BRAF, p53, and hTERT mutations [127], usually adopting an aggressive clinical course with distant metastases, RAI refractoriness, and mortality in a significant subset of patients has been reviewed elsewhere [34]. The identification of malignancy patterns of growth (solid, trabecular, or insular) is usually the first hint of the diagnosis of PDTC. Our systematic review showed that an insular pattern is more common than the other two, although they can coexist. According to the 5th edition of the WHO classification of tumors of endocrine organs, when a mixture of differentiated and poorly differentiated areas are incorporated in the same tumor, the least differentiated tumor component, even if non-predominant, should be recorded. However, it remains debatable whether the cut-off value for the poorly differentiated area should be reported in PDTC tumors. Based on our review, several cases had a multiconstituent histology of well-differentiated (mostly PTC and FTC) with poorly differentiated areas without elaborating on their extent [49,50,55,56,58,69,71,72,73,91,100,103,107,109,112,113]. Interestingly, the Japanese society of thyroid surgery emphasizes that the presence of a poorly differentiated component should be acknowledged as a distinct entity from DTC, independent of its histological extent within the tumor [128]. Studies have utilized the poorly differentiated proportion to distinguish PDTC and DTC with poorly differentiated areas, advocating its difference in terms of natural history and prognosis [91]. In the majority of studies in our review, the extent of the poorly differentiated area was not reported, and a PDTC definition was decided to address this component. A large number of patients with PDTC reviewed herein presented with locally advanced disease (T3 or T4, extrathyroidal extension, and nodal involvement) [40,44,46,47,49,50,51,54,55,57,58,60,61,62,64,67,68,69,70,71,73,74,75,76,77,78,80,81,82,83,84,85,86,87,88,90,91,92,94,97,98,99,100,101,102,107,108,109,111,112,113,114,115,116,117,119] and distant metastases at presentation or during later stages, mostly in the lungs and bones [40,41,42,43,44,45,46,48,49,50,51,54,55,57,58,60,61,62,63,66,67,68,69,70,71,72,73,74,75,76,80,81,82,83,84,85,87,88,89,90,91,92,93,94,95,97,98,101,102,103,106,107,108,109,110,111,112,114,116,117,119]. These findings imply that even in our case and despite the fact of the failure to have a biopsy during the progression of the disease the metastases originated from the poorly differentiated component.

### 5.4. Molecular Events in PDTC

The currently accepted model of TC oncogenesis is the multistep model. The fact that DTC can harbor foci of PDTC and ATC strengthens this hypothesis [29,129,130]. Progression from follicular cells to DTC is marked by activating mutations of the MAPK (mitogen-activated protein kinase) or/and PI3K/AKT (phosphatidylinositol 3-kinase/protein kinase B) pathways. Further progression to PDTC and ATC is characterized by additional mutations and genetic and epigenetic events that favor genetic instability and oncogenesis [29]. The development of PDTC is hypothesized to be secondary to this “multi-hit” process, with TERT promoter mutations being the most common molecular findings in PDTC (40%), with stepwise increases from PTC (9%) [131] to PDTC and ATC (65–73%) [132,133]. Concomitant mutations in RET/PTC, RAS, and BRAF promoters have been observed in advanced PTC and confer an unfavorable prognosis [134,135,136,137,138]. BRAFV600E is reported as the most common mutation, and its presence is associated with higher mortality [139], higher risk of recurrence [140], and loss of NIS (sodium/iodide symporter) expression [141]. However, this can also be observed in low-risk PTCs [142,143]. In our case report the presence of the BRAFV600E mutation correlated with a highly recurrent malignancy, in line with previous findings regarding the increased aggressiveness of BRAF-positive tumors. Interestingly, throughout our systematic review, the most commonly detected genetic events in PDTC were BRAF, RAS, and the TERT promoter. Additionally, for some of the cases, PTEN and/or TP53 alterations were identified (Table 1).

### 5.5. Survival

The modern criteria for PDTC were established in the 2007 Turin consensus proposal and have been reaffirmed by the 5th edition of the WHO classification of endocrine and neuroendocrine tumors [144]. Being morphologically and biologically intermediate between DTC and ATC, PDTCs usually exhibit more aggressive behaviors than DTCs, namely ETE and distant metastasis, coinciding with poorer outcomes [145]. Despite its rarity, PDTC accounts for most fatalities from non-anaplastic follicular cell-derived TC, holding an intermediate position between DTC and ATC in terms of five-year overall survival (OS), disease-specific survival (DSS), and disease-free survival. [146]. This is also depicted in our review, where few patients went disease-free [36,39,45,52,53,54,56,58,59,64,65,68,71,72,87,96,99,103,104,106,108,117,118]. Distant metastases represent the most significant cause of death in PDTC, with the majority of patients succumbing to the disease.

### 5.6. Chemotherapy

At present, there is no effective targeted chemotherapeutic regime for PDTC. Cisplatin/doxorubicin and carboplatin/paclitaxel are similar combinations that are used to modulate disease progression, and likewise, only a few of the revised studies used these schemes for select patients [42,57,60,78,97,114,119].

### 5.7. Tyrosine Kinase Inhibitors

The role of tyrosine kinase inhibitors is evolving as a promising approach for treating PDTC in the near future. Sorafenib and lenvatinib have been approved by the U.S. Food and Drug Administration (FDA) for progressive, recurrent, or metastatic follicular cell-derived RAIR TCs. This was based on two seminal phase 3 trials (DECISION and SELECT, respectively), which included a limited number of PDTC cases, with the respective subgroup analyses available in the appendix of the studies [147,148]. In fact, in the cases discussed herein, TKIs, especially lenvatinib and sorafenib, were widely used in metastatic disease [42,44,50,55,60,67,68,73,75,80,81,84,89,90,94,98,109,110,111]. The actual benefit in terms of patient survival remains to be seen, since, in some patients, control was obtained [42,44,64,67,73,80,81,84,97,110], while others succumbed to the disease [55,60,86,89,90,94,97,98,109,111]. In the sole cohort study including 8 PDTC patients who had all received lenvatinib, this TKI treatment achieved a median progression-free survival (PFS) of 12 months [94]. In our case, where lenvatinib was administered alternatively with sorafenib, due to their AEs, the combined PFS had reached 30 months.

While Sorafenib [147] and Lenvatinib [148] are still considered first-line treatments in advanced RAIR TCs, patients with the BRAFV600E mutation may benefit from treatment with BRAF-I once they have progressed on standard-of-care treatments. BRAF-Is (dabrafenib, vemurafenib) have been implemented for the management of TC [30,149]. BRAF-Is have not yet been FDA approved for the treatment of BRAF-mutant RAIR-DTC. A dabrafenib/tramatenib combination was recently FDA approved for the treatment of BRAF-mutated ATC [150], while a phase II randomized clinical trial studying the use of a BRAF-I (dabrafenib) and BRAF-I/MEK-I (dabrafenib/tramatenib) combination in BRAF-mutated DTC demonstrated high objective response rates and efficacy of both the single agent and the combination [35]. An ongoing global, multicenter, randomized, double-blind, placebo-controlled phase III study aims to evaluate the efficacy and safety of dabrafenib/trametinib in adult patients with locally advanced or metastatic BRAFV600E mutation-positive RAIR-DTC that have progressed following prior VEGFR targeted therapy. Patients are randomized to either dabrafenib/trametinib or a placebo and stratified based on the number of prior VEGFR targeted therapies (1 versus 2) and prior lenvatinib treatment (NCT04940052) [151]. In our BRAF V600E-positive case, due to drug intolerance and disease progression, a change from sorafenib/lenvatinib to the BRAF/MEK-I combination resulted in remarkable shrinkage of all lesions, leading to a further PFS of 24 months. A similar combination of dabrafenib/trametinib, as administered in our patient, was used to treat two PDTC patients, one of them presenting disease remission in one of the revised studies [92]. These data suggest potential benefits and a better tolerability profile of the dabrafenib/trametinib combination in PDTC patients, which should urge for further studies investigating appropriate sequelae of treatments. We could speculate that an earlier administration may increase the PFS in these patients. Another newly employed treatment approach in PDTC cases is the co-administration of lenvatinib and pembrolizumab. The combination of both TKI and classic immunotherapy achieved a median PFS of 17.7 months in two PDTC patients [60].

The critical limitations of targeted therapies are their major AEs profile, as in our case, that occasionally cannot be counteracted by dosede-escalation and/or adjunct agents and, most importantly, the development of escape mechanisms developed by the tumor. Although targeted therapies exhibited significant efficacy in our case, the related AEs were also notable. The balance between drug efficacy, management of AEs, and drug resistance is truly challenging. The fact that the occurrence of certain AEs has been proposed as a predictive response marker (hypertension correlated with improved outcomes on lenvatinib treatment) [152] calls for the timely implementation of preventive strategies to avoid toxicity and prolong therapeutic use.

There are certain limitations of our systematic review that should be addressed. Although we narrowed down our search to the time period after 2017, we did not evaluate the histopathological reviews of the presented studies to inspect their unanimity. We relied on the diagnosis of PDTC presented in each study, whether a detailed pathology review was provided or not. Furthermore, since the majority of the studies did not report the exact percentage of the PDTC component in DTC, we decided to include any relevant study with any component of poorly differentiated area in the pathology review. Additionally, despite the exclusion of pediatric populations, we decided to include a few cohort studies that also included, among others, minor patients [54,58,74,91,112,113], since they could shed light on the characteristics of PDTC in a wider population.

## 6. Conclusions

PDTC is a histological entitiy that isclassified as intermediate in the spectrum of TC in terms of biological behavior and prognosis. In terms of therapeutics, it poses a challenge, as itis frequently not RAI avid, limiting current therapeutic options. However, with insight into molecular mechanisms rapidly increasing, TC therapy should be individualized on a case-by-case basis, employing molecular diagnostics in everyday practice. Molecular testing is now warranted to plan the treatment strategy for both anaplastic and PDTC. The detection of BRAF mutations, RET fusions, or NTRK rearrangements is of utmost importance for diagnosis and treatment, considering the reported promising results of novel targeted therapies. Herein, we report a case of metastatic mixed PDTC and present the efficacy of the sequential administration of targeted drugs. The most favorable therapeutic sequence for each individual patient or group of patients remains to be further elucidated as new evidence comes to light.

## Figures and Tables

**Table 1:** Characteristics of the included studies.

Study	*n*	M/F	Age	Histological Combinations	ETE	Dm	Ln	Surgery	RAI	Systematic Treatment	Outcome	Molecular
Abdellaoui, 2022 [38]	1	1/0	27	Insular, solid	0	0	0	Rt HTmy (first), TTmy	1	0	AND	NA
Abu Rumman, 2021 [39]	1	1/0	23	Insular, solid, trabecular (thyroglossal cyst)	*	0	0	Sistrunk procedure (first), TTmy	1	0	AND	NA
Agarwal, 2024 [40]	39	1/2.9	55 (median)	Insular (32), solid (25), trabecular (18), PTC (2), FTC (14)	67.7%	57%	45.1%	TTmy (+lateral dissection 10)	25.6% (multiple)	0	73% survival (12 m)	BRAF (5), NRAS (9)
Ahmadian, 2024 [41]	1	1/0	71	PTC (thyroid), PDTC (pancreas, liver)	0	Liver, pancreas, peritoneum, (adrenal glands)	NA	TTmy (+ND)	1	NA	NA	AGAP3: BRAF fusion
Alshehri K, 2022 [42]	1	0/1	56	PDTC	1	Lung, sternal osseous	0	TTmy (+central, Rt ND)	1	EBRT (15) + (paclitaxel, carboplatin, doxorubicin, sorafenib, lenvatinib)	AWD	HRAS-BCORL1
Altiner, 2023 [43]	1	0/1	70	PDTC	0	Lung, rib, orbital	0	TTmy, rid resection	1	Paclitaxel	AWD	NA
Alzahrani, 2022 [44]	1	0/1	43	Insular	1	Lung, (adrenal)	1	PTmy	0	Lenvatinib	AWD	KRAS+ PIC3CA (missense), PICR1 (insertion), FH (nonsense)
Ambre, 2022 [45]	1	0/1	74	Insular, trabecular	0	Mandible	0	TTmy, hemi-mandibulectomy	0	0	AND	NA
Atif, 2018 [46]	1	0/1	56	PDTC	0	Lung, bones	1	TTmy (+CND)	1			KRAS
Bellini, 2021 [47]	8	3/5	54–85	PDTC, oncocytic	5/6	NA	3/8	TTmy (6), nodule removal (2), lymphadenectomy (5)	NA	NA	NA	NA
Bertrand, 2019 [48]	1	0/1	53	PTC (thyroid), PDTC–trabecular (uterus)	0	Lung, bones, uterus	0	TTmy (+CND)	2	NA	AWD	NA
Beute, 2024 [49]	7	3/4	30–82	PDTC (7/7), hobnail (1/7), tall-cell (1/7)	5/7	2/7	2/3	TTmy (4/7), STTmy (2/7), lymphadenectomy (6/7)	1/1	EBRT (1/1)	NA	BRAF (1/1)
Bichoo, 2019 [50]	142	1/2.1	50.9 (mean)	PDTC (27/142), PTC with PDA (27/142), FTC with PDA (88/142)	51/142	75/142	43/142	TTmy (136/142), near total (1/142), HTmy (1/142), debulking (4/142), lymphadenectomy (53/142), resection of metastasis (20/142)	110/142	EBRT (30/142), Sorafenib (3/142)	64% (G1), 85% (G2), 62% (G3) DOD. From those alive: 66% (G1), 50% (G2), 39% (G3) AWD	NA
Brijmohan, 2023 [51]	1	1/0	68	PDTC (insular, solid, trabecular), PTC	1/1	Liver, bones, scalp	1/1	TTmy (+ND)	1/1	NA	NA	NA
Ching, 2018 [52]	1	0/1	44	PDTC (insular, trabecular)	0	0	0	Rt Tmy (first), TTmy	NA	NA	AND	NA
Choi, 2021 [53]	1	0/1	61	PDTC (insular, solid, trabecular)	0	0	0	TTmy	1	NA	AND	TERT promoter point mutation
Choi, 2020 [54]	21	7/14	15–78	PDTC (insular, trabecular), FC (15/21), PTC (5/21),	21/21	4/21 (lung, bones)	0	TTmy (8/21), CTmy (10/21)	19/21	0	AND (16/21) AWD (5/21)	NA
Colombo, 2022 [55]	1	1/0	35	PDTC, FTC	1	Lung, liver, adrenal	1	TTmy, thoracic surgery	1	Sorafenib	DOD	PTEN, p53
Corean, 2019 [56]	1	0/1	29	CMVPTC, PDTC	0	0	0	TTmy	0	NA	AND	APC
Das, 2021 [57]	1	1/0	58	Poorly differentiated follicular carcinoma with rhabdoid phenotype	1/1	Lung	NA	Tracheostomy only	NA	Cisplatin, endoxan, doxorubicin	AWD	NA
de la Fouchardière, 2018 [58]	104	40/64	12–91	Insular (93/104), solid (11/104), trabecular (17/104), PTC (59/102), FTC (29/102), Hurthle (14/102)	40/100	17/98	11/98	TTmy (101/104), ND (36/104)	99/104 (1–6 cycles)	EBRT (9/104), TKIs (21/104)	AND (36/104), AWD (33/104), DOD (35/104)	TERT promoter (24/63), BRAF (3/38), RAS (10/38)
Dettmer, 2023 [59]	1	0/1	44	CMTC, PDTC	0	0	0	Rt Tmy (first), TTmy	1	0	AND	APC, TERT promoter, PIC3CA
Dierks, 2021 [60]	2	NA	49/63	PDTC	2/2	2/2 (bone, lung, liver, kidney)	2/2	TTmy (2/2), ND(1/2)	1/2 (2 cycles)	EBRT (1/2), cisplatin/doxorubicin and carboplatin/paclitaxel (1/2), lenvatinib + pembrolizumab (2/2)	DOD (2/2)	TERT promoter and PTEN (2/2)
Elshafie, 2023 [61]	1	0/1	60	PDTC	NA	Bones, lung	1/1	TTmy (+centra/left lateral ND)	1	EBRT	AWD	NA
Farahmandfar, 2020 [62]	1	1/0	70	PTC, PDTC	NA	Parotid, lung, pericardium	1/1	TTmy (+CND)	1	EBRT, chemo (NA)	DOD	NA
Feffer, 2017 [63]	1	0/1	55	PDTC (insular)	NA	Sphenoid bone	0	0	0	EBRT	AWD	NA
Gay, 2019 [64]	1	0/1	81	PDTC with focal squamous differentiation	1/1	0	0	TTmy	1	EBRT, lenvatinib	AND	NA
Gazeu, 2020 [65]	1	0/1	43	PDTC (solid), oncocytic, follicular component	0	0	0	Rt Tmy (first), TTmy	1	0	AND	0
Gill, 2023 [66]	1	0/1	46	PDTC	0	SVC tumor thrombus	1	TTmy	3	EBRT	AWD	NA
Goto, 2018 [67]	1	0/1	75	PDTC, PTC	NA	Lung	NA	TTmy, ND	1	Lenvatinib, sorafenib	AWD	NA
Grawe, 2021 [68]	47	21/26	57 ± 19	PDTC	9/47	18/47	14/47	TTmy	1 (0–3)	Lenvatinib/sorafenib (2/47), EBRT	AND (18/47), AWD (15/47), DOD (14/47)	NA
Gubbiotti, 2023 [69]	65	29/36	21–85	PDTC, PTC (52/65), FTC(13/65)	31/65	19/65 (lung, bones, brain)	11/65	TTmy (53/65), lobectomy first (11/12)	42/65	Chemo (6/65), EBRT (4/65)	DOD (11/65)	HRAS (2/8), NRAS (2/8), BRAF (1/8), TERT promoter (1/8), p53 (2/8), PAX8: PPARy rearrangement (2/8), NSD3:: NUTM1 fusion(1/8)
Gülbahar Ateş, 2024 [70]	1	0/1	66	PDTC (solid, insular, trabecular)	1/1	Lung, bones, tumor thrombus	0	Neck surgery	0	EBRT	AWD	NA
Hu, 2022 [71]	1	0/1	35	PTC, PDTC	NA	Breast, neck	1/1	Endoscopic Tmy (first), TTmy (+bilateral CND), local tumor excision, modified radical ND, partial mastectomy	0	Adjuvant therapy (?)	AND	0
Ieni, 2021 [72]	1	1/0	69	FTC, PDTC	0	Kidney	0	Partial nephrectomy, TTmy	0	0	AND	NA
Iravani, 2019 [73]	3	0/3	45–61	FTC, PDTC	3/3	3/3 (lung, neck, muscle, bone)	1/3	TTmy (3/3), lymphadenectomy (1/3)	3/3	Sunitinib, sorafenib (1/3), trametinib (3/3), lenvatinib(2/3)	AWD (3/3)	NRAS (3/3)
Isaev, 2022 [74]	91	35/56	16–93	PDTC	63/91	38/91	37/91	TTmy (55/91), extended TTmy (25/91), lobectomy (9/91), subtotal (2/91), ND (59/91)	67/85	EBRT (7/85), systemic therapy (11/89)	AWD (40/91), DOD (27/91)	NA
Kalshetty, 2018 [75]	3	2/1	32–51	PDTC (3/3), PTC (1/3), focal cribriform (1/3)	2/3	3/3(bone, pleura, neck, lung)	1/3	TTmy (3/3), lymphadenectomy (2/3), tumor resection	2/3	EBRT (2/3), sorafenib (1/3)	DOD (1/3), AWD(2/3)	NA
Kersting, 2021 [76]	1	1/0	38	PDTC	0	Lung	1/1	NA, metastasectomy	NA	TKIs	AWD	NA
Khetrapal, 2018 [77]	1	0/1	42	PDTC	1/1	NA	1/1	TTmy, CND	NA	NA	NA	NA
Kim, 2023 [78]	1	1/0	34	PDTC (solid), SCC	1/1	Lung (SCC)	NA	TTmy, ND, lobectomy	NA	Paclitaxel, cisplatin, and etoposide	DOD	ATRX (c.6793G>T), TP53 (c.377A>G) MYCL (c.332G>T)
im, 2023 [79]	1	0/1	67	PDTC (neck)	0	0	0	lobectomy + CND (for benign lesion), neck excision	NA	NA	AWD	NA
Kunte, 2022 [80]	23	14/9	39–89	PDTC (23/23), PTC(7/23), FTC(7/23)	18/13	7/23	8/23	TTmy (19/23), lymphadenectomy (8/19)	13/23	EBRT (2/19), TKIs (sorafenib, lenvatinib, sorafenib, and pazopanib)(6/23)	DOD (12/23)	NRAS, TERT promoter (1/2)
Kut, 2020 [81]	1	1/0	60	FTC, PDTC (insular)	1/1	Lung	1/1	TTmy, lymphadenectomy, neck surgery	1	EBRT, TKIs (lenvatinib, pazopanib), pembrolizumab	AWD	TERT promoter
Laforga and Cortés, 2019 [82]	1	1/0	76	OV–PDTC	NA	Lung, spleen, bones, liver	1/1	TTmy, bilateral ND	1	NA	DOD	NA
Leboulleux, 2019 [83]	1	0/1	59	PDTC	1/1	Lung, bone	1/1	0	0	Dabrafenib and trametinib	AWD	BRAF-K601E mutation
Lee, 2021 [84]	1	1/0	76	PDTC	1/1	Lung	1/1	0	0	Carboplatin, EBRT, pembrolizumab, lenvatinib, rametinib, dabrafenib	AWD	BRAF T599_V600insT, CDKN2A/B loss, loss of MTAP exons 2–8 and TERT promoter mutation
Lukovic, 2021 [85]	45	24/21	35.5–83.6	OV–PDTC	6/45	16/45	3/16	TTmy (21/45), staged Tmy (24/45)	39/45	EBRT (4/45), VEGF	NA	NA
Molinaro, 2019 [86]	1	0/1	65	PTC, PDTC with squamous cells	1/1	0	0	TTmy	1	Lenvatinib	DOD	BRAF V600E
Morvan, 2022 [87]	1	0/1	58	PDTC	1/1	Bone, IJV tumor thrombus	0	TTmy + removal infiltrated portion IJV, ND	1	0	AND	NA
Nagaoka, 2023 [88]	1	1/0	70	PDTC	1/1	Bones	0	TTmy	1	0	AWD	NA
O’Donohue, 2022 [89]	1	1/0	79	PDTC	0	Liver (port hepatis), bones	0	TTmy	0	Lenvatinib	DOD	
Oh, 2024 [90]	1	0/1	50	PDTC	1/1	Lung, liver, bones	1/1	TTmy	1	EBRT, lenvatinib, nivolumab and ipilimumab, cabozantinib, temsirolimus, nivolumab + relatinib	DOD	PTEN L194fs and TP53 F270S
Panchangam, 2022 [91]	61	1/1.3–1.6	16–81	PDTC, PTC with PDA	59–73%	19–41%	50–55%	TTmy (52/61), near Tmy (5/61), debulking (4/61)	29–65%	EBRT (7/61), chemo(1/61)	NA	NA
Peng, 2022 [92]	2	0/2	72–73	PDTC, poorly differentiated squamous cell carcinoma	2/2	1/2(lung)	1/2	TTmy + ND(Hx PTC)	3(Hx PTC)	Anlotinib (1/2), dabrafenib, and trametinib (2/2)	DOC (1/2), AWD (1/2)	BRAFV660E (1/2), TERT promoter (1/2)
Pinto, 2019 [93]	1	0/1	71	PDTC with a focal papillary and follicular pattern	NA	Lung, bones	0	TTmy	0	0	DOD	NA
Prete, 2024 [94]	2	2/0	68–69	PDTC	1/2	Lung, IJV + SVC tumor thrombus	1/2	TTmy (1/2), thrombectomy (1/2), lymphadenectomy(2/2)	0	Lenvatinib (1/2)	DOC (1/2), AWD(1/2)	NA
Purbhoo, 2023 [95]	1	0/1	51	PDTC (solid, insular)	0	Bone, lung, pituitary gland	0	TTmy + CND	NA	NA	AWD	NA
Raffaelli, 2023 [96]	1	0/1	72	PDTC	1/1	0	1/1	TTmy + central/ lateral ND	NA	NA	AND	NA
Roque, 2023 [97]	8	3/5	34–67	PDTC	NA	7/8	NA	Surgery (6/8), metastasis excision (2/8)	6/8	EBRT (4/8), paclitaxel and carboplatin (1/8), other TKIS(3/8), lenvatinib(8/8)	AWD (5/8), DOD(3/8)	NA
Temperley, 2023 [98]	1	1/0	69	PDTC	1/1	1/1(liver, bones, hilar LN)	0	0	0	Lenvatinib, cabozantinib, EBRT	DOD	NA
Sato, 2017 [99]	1	0/1	64	PTC, PDTC	1/1	0	0	total Rt thyroid lobectomy isthmectomy, resection + CND	1	NA	AND	NA
Schopper, 2017 [100]	1	1/0	49	Conventional PTC, follicular variant of papillary carcinoma, columnar cell carcinoma, clear cell papillary carcinoma, PDCT	1/1	0	1/1	TTmy + L ND	NA	NA	NA	KRAS, BRAF (PDTC)
Shakeri, 2020 [101]	1	0/1	58	PDTC	NA	Lung, brain	1/1	TTmy + ND	1/1	NA	NA	NA
Sonavane, 2023 [102]	1	1/0	39	PDTC	1/1	Brain, bones, lung	1/1	TTmy + CND	3	EBRT	AWD	NA
Suehiro, 2021 [103]	6	1/5	21–67	PDTC (6/6), PTC (2/6), squamous differentiation (1/6), signet ring differentiation (1/6)	NA	Axillary lymph nodes	NA	TTmy (5/6), CTmy (1/6), ND (6/6), axillary node dissection (6/6)	NA	NA	AWD (2/3), AND (1/3)	NA
Sugawar, 2023 [104]	1	0/1	36	PDTC (trabecular)	NA	NA	NA	left lobectomy (first), TTmy	1	0	AND	NA
Sukrithan, 2023 [105]	1	1/0	70 s	PDTC	NA	1/1	NA	TTmy	1	EBRT, lenvatinib	AWD	NA
Suman and Basu, 2018 [106]	1	0/1	77	PDTC, FTC (bones)	0	Bones	0	TTmy + Rt CND	2	0	AND	NA
Thiagarajan, 2020 [107]	35	11/24	22–77	PDTC (solid (10/35), insular (11/35), trabecular (1/35), mixed (6/35))	12/35	18/35	9/35	completion Tmy (7/35), TTmy (27/35), thyroid bed exploration (1/35), ND(21/53)	35/35	EBRT (16/35)	AND (23/35), AWD (10/35), DOD (2/35)	NA
Thompson, 2023 [108]	24	11/13	58(mean)	PDTC (24/24), PTC (13/24), metastatic SCC to thyroid gland (1/24)	9/24	5/24	6/24	Surgery (lobectomy, Tmy, and/or CTmy) (24/24)	18/24	EBRT (5/24), chemo (1/24)	AND (15/24), AWD (3/24), DOD (5/24), DOC(1/24)	NRAS (3/13) TERT+ NRAS (1/13), TERT + NRAS + KAT6B (1/13) NRAS + MUTYH (1/13); NRAS + PALB2 (1/13) PTENdeletion (1/13), WHSC1L1::NUTM1 (1/13), PAX8::PPARγ (1/13)
Toyoshima, 2021 [109]	1	0/1	63	Oncocytic carcinoma, PTC (classic and hobnail component), PDTC	0	Lung, liver	1/1	TTmy, bilateral LN dissection	1	EBRT, sorafenib	DOD	NA
Tsuji, 2018 [110]	1	0/1	26	CMV–PTC (thyroid), CMV–PTC, PDTC (lung)	NA	Lung	0	TTmy, cervical lymph ND, Rt partial lobectomy	1	Sorafenib	AWD	NA
Uchida, 2019 [111]	1	0/1	73	PDTC	NA	Lung, pleura	1/1	0	1/1	Lenvatinib	DOD	NA
Wan, 2023 [112]	94	34/55	8–85	PDTC (94/94), PTC only (20/94), FTC only (17/94), other (34/94)	83/94	23/94	78/94	Tmy (73/95), ND(29/94)	23/94	EBRT (26/94), chemo (22/94)	Median OS (m) (min–max) 33(1–170)	NA
Xu, 2023 [113]	210	101/109	5–87	PDTC, oncocytic component (79/210)	132/210	NA	39/210	lobectomy/HTmy (38/210), TTmy/STmy (172/210)	157/200	EBRT (90/199), TKIs (67/209), chemo (37/199)	NA	BRAF (6/87), NRAS (35/87), HRAS (4/87), KRAS (3/87), RET (1/87), PPARG (1/60), ALK (1/87), TERT (43/87), PTEN (15/87), TP53 (15/87), EIF1AX (11/87), NF 1(6/87), RBM10 (5/87), ATM (4/87), DNMT3A (4/87), STK11 (4/87), ARID1A (4/87)
Xue, 2017 [114]	5	2/3	43–76	PDTC	5/5	2/5 (lung)	5/5	unilateral Tmy (1/5)	NA	IMRT (5/5), gemcitabine (1/5), cisplatin (1/5) paclitaxel and cisplatin (4/5)	AWD (2/5), DOD (3/5)	NA
Yasuoka, 2017 [115]	1	0/1	35	PDTC (solid, trabecular, microfollicular)	1/1	0	0	L HTmy (+ND), completion Tmy (+CND)	1	EBRT	AND	NRASgene
Yin, 2023 [116]	206	98/108	57.9 ± 15.9	Insular	158/200 (T3 + T4)	53/202	66/192	193/204	155/206	Chemo (22/206)	90.3% (1year-OS), 82.0% (2year-OS), 62.2% (5 year-OS), and 42.5%(10 year-OS)	NA
Yu, 2017 [117]	18	5/13	40–75	PDTC insular (8/18), trabecular (7/18), solid (3/18)	8/18	5/18 (lung (3/5), bone (2/5))	5/18	TTmy (+neck dissection) (5/18), TTmy only (5/18), subtotal + completion Tmy (2/18), lobectomy completion (1/18), tumor debulking (4/18)	8/18	0	AND (6/18), AWD (9/18), DOD (3/18)	NA
Yuang, 2023 [118]	1	1/0	66	PDTC (solid/trabecular), FTC	NA	0	0	Rt lobectomy + isthmectomy	0	0	AND	NA
Zhang, 2023 [119]	2	1/1	<60 (1/2), >60 (1/2)	PDTC, poorly differentiated squamous cell carcinoma	1/2	1/2 (lung, bone, brain)	2/2	0	EBRT (1/2)	nab-paclitaxel + carboplatin (2/2), toripalimab (2/2)	AWD (1/2), DOD (1/2)	NA
PRESENT CASE	1	1/0	45	PDTC, hobnail, tall-cell	1/1	1/1 (brain, bone, sinus)	1/1	TTmy (+neck dissection, completion ND)	2	Lenvatinib, sorafenib, trametinib/dabrafenib	DOD	BRAF

Note: AND: alive without disease; AWD: alive with disease, CMTC: Cribriform—morular thyroid carcinoma; CMV-PTC: Cribriform-morular variant of papillary thyroid carcinoma; CND: central neck dissection; CTmy: completion thyroidectomy; Dm: distant metastases; DOC: died of other causes; DOD: died of disease; EBRT: external beam radiation therapy; ETE: extrathyroidal extension; FTC: follicular thyroid carcinoma, HTmy: hemithyroidectomy; IJV: internal jugular vein; IMRT: Intensity-modulated radiation therapy; L: left; Ln: lymph nodes; OV-PDTC: oncocytic variant poorly differentiated thyroid carcinoma, PDA: poorly differentiated areas, M/F: male-to-female, N: number of patients, NA: not applicable or, specifically, in molecular section, BRAF negative when performed; PDTC: poorly differentiated thyroid carcinoma, PTC: papillary thyroid carcinoma; PTmy: partial thyroidectomy; RAI: radioactive iodine; Rt: right; SVC: superior vena cava, TKI: tyrosine kinase inhibitor; Tmy: thyroidectomy; TTmy: total thyroidectomy, * thyroglossal cyst.

## Data Availability

The data will be available upon reasonable request.
